# Evolutionary Reorganization of Transcriptomic Architecture Across a UVB Tolerance Gradient in Fish

**DOI:** 10.1002/ece3.74298

**Published:** 2026-09-03

**Authors:** Camilo Valdivieso, Miguel Luis Allende

**Affiliations:** ^1^ Center for Genome Regulation Santiago Chile; ^2^ Laboratorio de Genómica y Genética Molecular, Departamento de Biología, Facultad de Ciencias Universidad de Chile Santiago Chile

**Keywords:** adaptive evolution, network biology, network reorganization, transcriptomic architecture, UVB radiation

## Abstract

Environmental stressors such as ultraviolet radiation impose strong selective pressures on organisms, yet how adaptation to such stressors shapes transcriptomic responses at the network level remains poorly understood. Although stratospheric ozone is recovering globally, substantial regional variation in UV exposure persists, particularly in high‐altitude environments where extreme UV levels can occur. Here, we compared three fish models representing distinct biological responses to UVB exposure: wild‐type zebrafish ( *Danio rerio* ), a melanin‐deficient zebrafish mutant (*nacre*) lacking a major protective mechanism against UVB damage, and the high‐altitude Andean killifish *Orestias ascotanensis* , a species naturally exposed to extreme UVB radiation. Together, these models define a gradient spanning physiological protection, impaired protection, and evolutionary adaptation to UVB stress. Using RNA‐seq and protein–protein interaction networks, we show that transcriptomic responses differ markedly across this gradient. Wild‐type and *nacre* zebrafish exhibited relatively limited transcriptomic changes (∼2%–2.4% of genes changing), whereas *O. ascotanensis* displayed a large‐scale and highly coordinated response (∼21.6% of genes changing) characterized by functionally specialized networks enriched in DNA repair pathways. These differences involved not only transcriptomic magnitude but also marked reorganization of transcriptomic architecture. Integration with positive selection analyses revealed that positively selected genes were concentrated within highly interconnected regions of transcriptomic networks, consistent with adaptation involving network reorganization. Furthermore, ortholog‐based analyses suggest that adaptive responses involve differential reorganization of a conserved functional background. Together, our results support a model in which adaptation to environmental stress is associated with the reorganization of conserved transcriptomic networks across physiological and evolutionary contexts, providing a systems‐level perspective on the molecular basis of adaptation.

## Graphical abstract

Environmental stress can drive adaptation through changes in transcriptomic organization. Across a UVB tolerance gradient in fish, adaptive responses were associated with increasingly coordinated and functionally specialized transcriptomic networks built upon a conserved functional background.

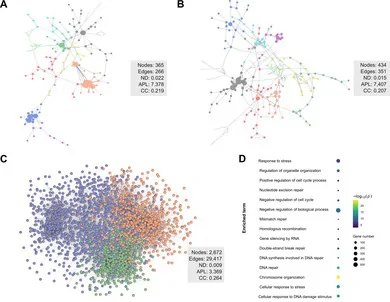

## 1 Introduction

The ultraviolet B (UVB) radiation emitted from the sun is strongly attenuated by the ozone layer in the atmosphere, but a fraction of UVB photons reaches the Earth's surface. Following implementation of the Montreal Protocol, stratospheric ozone has been recovering, although recovery is spatially heterogeneous. Accordingly, changes in surface UV radiation are also regionally variable, with recent trends generally being small at low and mid‐latitudes but increasing at some locations and decreasing at others, depending on ozone, aerosols, clouds, and other environmental factors (Bernhard et al. 2023; WMO 2022). Climate change can further influence stratospheric ozone and atmospheric circulation, contributing to complex interactions among climate, ozone, and surface UV radiation (Bernhard et al. 2023; WMO 2022). In particular, high‐altitude environments characterized by low atmospheric attenuation and high solar irradiance can experience extreme UV exposure (Cordero et al. 2018). In aquatic environments, UVB radiation can penetrate down to depths of 1 m (Fleischmann 1989), and can affect several fish behaviors such as communication, motility, foraging, and mate selection, among others (Williamson and Rose 2010). Furthermore, adaptive behaviors have even been reported, such as UVB avoidance in juveniles of coho salmon (Kelly and Bothwell 2002). As a mutagenic agent, UVB radiation imposes a strong selective pressure by inducing direct DNA damage through pyrimidine dimer formation, as well as oxidative stress, *p53* activation, and an inflammatory response (de Gruijl 2002; Besaratinia et al. 2005; Cadet et al. 2015). In response, fish have evolved multiple cellular strategies to cope with UVB‐induced damage, including the synthesis and reorganization of protective pigments such as melanin (Mueller and Neuhauss 2014; Neuffer and Cooper 2022), the activation of DNA repair pathways involving chromatin remodeling by the nucleotide excision repair (NER) pathway (Palomera‐Sánchez and Zurita 2011), and transcriptomic responses to UVB exposure (Yang et al. 2014; Alves and Agusti 2022), associated with phenotypic plasticity (West‐Eberhard 1989; Goldstein and Ehrenreich 2021). However, how these responses are organized at the transcriptomic network level, and how their architecture may differ between physiological protection and long‐term evolutionary adaptation, remains poorly understood.

In fish, transcriptional responses to UVB exposure have been studied in model systems such as *Danio rerio* (Hamilton, 1822), focusing on stress and inflammatory response genes (Aksakal and Ciltas 2018), and through transcriptomic approaches in species such as *Xiphophorus maculatus* (Yang et al. 2014) and *Sparus aurata* (Alves and Agusti 2022). These studies have primarily characterized transcriptional responses to UVB exposure by identifying differentially expressed genes and affected biological pathways. However, whether UVB responses differ in their network‐level organization among organisms with contrasting physiological and evolutionary histories of UVB exposure remains poorly understood. Network‐based approaches, including protein–protein interaction (PPI) networks inferred from transcriptomic data, provide a framework to explore higher‐order organization (Markowetz and Spang 2007; Karlebach and Shamir 2008). The architecture of transcriptomic responses can also be characterized using network‐based approaches, including metrics such as network density, clustering coefficients, and average path length (Pavlopoulos et al. 2011), as well as node centrality parameters such as degree and betweenness (Newman 2006) that identify highly connected genes. These architectural properties provide insights into the organization and functional specificity of transcriptomic responses. In guppies, for instance, transcriptomic architecture analysis revealed hub genes associated with the evolution of sociability and schooling behaviors (Corral‐Lopez et al. 2023). Thus, a network‐level comparison may reveal aspects of environmental adaptation that are not apparent from differential gene expression alone.

Extreme environments provide natural systems to study adaptation. Recently, two UVB radiation hotspots were reported in the South American Altiplano region, in northern Chile (Cordero et al. 2018), partially overlapping with the geographic distribution of the high‐altitude teleost killifish genus *Orestias* (Parenti 1984). These species have been isolated in highland aquatic systems since the end of the primary uplift of the Andes Mountain range between 65 and 34 Mya (Gregory‐Wodzicki 2000; Garzione et al. 2008; Morales et al. 2024) and, since then, have experienced exposure to extreme environmental conditions. In particular, genome sequencing and transcriptomic approaches have identified positively selected genes associated with DNA repair in natural populations of *Orestias ascotanensis* Parenti (1984), providing evidence for evolutionary adaptation to high UVB exposure (di Genova et al. 2022). However, whether this evolutionary history is reflected in the network‐level organization of the UVB‐induced transcriptomic response remains unknown.

In contrast, the zebrafish *D. rerio* is a well‐established genetic and developmental model well suited to studying physiological mechanisms against UVB, particularly the role of skin pigmentation (Mueller and Neuhauss 2014). Importantly, mutant lines lacking melanophores, such as *nacre* (*mitf1a*−/−) (Lister et al. 1999), provide a controlled system to examine UVB responses when a major physiological protective mechanism is impaired. Together with *O. ascotanensis* , these models provide contrasting biological contexts in which to examine UVB‐responsive transcriptomic organization, ranging from physiological protection and impaired protection to long‐term adaptation to extreme UVB environments.

Here, we performed RNA‐seq analyses across these three fish models, integrating differential gene expression, PPI networks, and functional enrichment analyses. We use “physiological response” to refer to short‐term transcriptional changes induced by UVB exposure within an organism, whereas “evolutionary adaptation” refers to heritable changes accumulated across generations that increase fitness under persistent environmental conditions. This comparative framework allowed us to evaluate how physiological and evolutionary processes shape the transcriptomic architecture of responses to UVB radiation. In this study, transcriptomic architecture refers to the organization, connectivity, and functional specialization of transcriptomic responses inferred from PPI networks derived from differentially expressed genes, rather than to the direct reconstruction of gene regulatory networks.

Accordingly, we hypothesized that the organization of the UVB‐induced transcriptomic response differs across physiological states of protection and evolutionary adaptation. We expected melanin‐based protection in wild‐type zebrafish to buffer UVB‐induced molecular damage, resulting in a comparatively limited transcriptional response, whereas the absence of melanization in *nacre* would increase the need for transcriptional responses to UVB. In contrast, we expected the long‐term UVB exposure experienced by *O. ascotanensis* to be associated with a more functionally specialized and highly organized transcriptomic architecture. We therefore predicted that the three models would differ not only in transcriptomic magnitude but also in network organization and functional specificity.

## 2 Materials and Methods

### 2.1 Zebrafish Lines and Sampling Sites

All procedures were approved by the Ethical Committee of Universidad de Chile (CICUA). The table 5 zebrafish line was used as the control genetic background (wild‐type), while the *nacre* mutant line (*mitf1a*−/−) was selected to evaluate the contribution of melanin to the response to UVB radiation. Both lines were maintained at the Faculty of Sciences facilities of Universidad de Chile. *O. ascotanensis* individuals were captured using hand nets at Spring 6, Salar de Ascotán, Región de Antofagasta (21°29′52.9″ S; 68°15′24.4″ W), and were exposed to the experimental conditions on the same day. Sampling of wild *O. ascotanensis* was conducted under Research Fishing Authorization Resolution Exempt No. E‐2020‐069, issued by the Chilean Undersecretariat for Fisheries and Aquaculture (SUBPESCA).

### 2.2 UVB Exposure Experiment, Skin Total RNA Isolation, and Transcriptome Sequencing

Groups of three adult female fish (*n* = 3 per experimental group) were exposed to a 10:14‐h light: dark photoperiod. Only female individuals were used to minimize potential sex‐related variation in transcriptomic responses. In *O. ascotanensis* , where sexual dimorphism is not readily apparent, larger individuals were preferentially selected because they are more likely to be females, and sex was subsequently confirmed at dissection based on gonadal morphology. Control fish were exposed to white light, whereas treatment fish were exposed to UVB radiation during the light phase. UVB exposure was performed using a portable exposure chamber equipped with four Philips TL20W/12 UV‐B fluorescent lamps. The chamber was constructed based on the experimental configuration described by Yang et al. (2014), but adapted for the present experimental design and field use. The distance between the lamps and the water surface was 30 cm. Fish were maintained in transparent 5‐L tanks (267 × 207 × 140 mm; L × W × H) containing approximately 10 cm of water depth. The same tanks were maintained throughout the experiment, and three‐quarters of the water volume was replaced daily. Zebrafish were maintained in system water (pH 8.0, conductivity 900 μS/cm, temperature 28°C), whereas *O. ascotanensis* were maintained in water collected from the spring at the sampling locality (pH 8.01, conductivity 5200 μS/cm, source‐water temperature 22.2°C). Both water sources were continuously aerated using air pumps. Within each species, control and UVB‐exposed fish were maintained under the same water conditions throughout the experiment.

UVB irradiance inside the exposure chamber was measured using a GENERAL UV513AB UVAB Digital Light Meter (280–400 nm). Measurements were performed with all four lamps operating and at multiple positions adjacent to the experimental tanks, yielding an irradiance of approximately 1500 μW/cm^2^ (15 W/m^2^) with no appreciable spatial variation within the chamber. The four lamps provided continuous illumination across the entire area occupied by the experimental tanks, with no dark zones within the exposure chamber. The sensor was positioned outside the water and adjacent to the tanks. Based on the measured irradiance at the position of the tanks and the 10‐h exposure period, the incident UVB dose was calculated as 540 kJ/m^2^ per exposure cycle. Fish underwent three consecutive daily exposure cycles, each consisting of a 10‐h UVB exposure followed by a 14‐h dark period, resulting in a cumulative calculated incident dose of 1620 kJ/m^2^. This repeated exposure regime was selected to maintain a consistent 10:14‐h light: dark cycle while providing a reproducible UVB challenge over multiple exposure periods, rather than delivering the entire calculated dose as a single continuous exposure.

Yang et al. (2014) demonstrated transcriptional responses in adult fish exposed to a UVB irradiance of 12.2 W/m^2^, with responses detected across doses ranging from 8 to 32 kJ/m^2^. Our experimental irradiance was of the same order of magnitude (15 W/m^2^), while the longer and repeated exposure regime was designed to provide a robust and reproducible UVB challenge for comparative transcriptomic analysis rather than to reproduce the acute exposure regime of Yang et al. (2014).

Both control and UVB‐exposed fish were maintained at 25°C throughout the experiment. This temperature was selected as an intermediate experimental condition between the maintenance temperature of zebrafish (28°C) and the temperature of the spring water at the *O. ascotanensis* sampling locality (approximately 22°C). Zebrafish were transferred from their maintenance tanks to the experimental tanks, whereas *O. ascotanensis* were exposed in the field using the portable chamber transported to the locality nearest to their natural habitat (Ollagüe, approximately 30 km from the sampling site). Thus, wild individuals were not transported to the laboratory or subjected to prolonged laboratory acclimation before UVB exposure. Following completion of the third exposure cycle, fish were euthanized and skin tissue was immediately collected for RNA extraction.

After the exposure regime, individuals were submerged in an overdose of anesthetic solution (tricaine methanesulfonate, Merck). Approximately 10 mg of dorsal skin tissue was dissected from each individual, homogenized, preserved in RNAlater solution (Thermo Fisher Scientific), and stored at −80°C until RNA extraction. Total RNA was isolated from skin tissue using TRIzol reagent (Thermo Fisher Scientific), followed by phenol: chloroform extraction (Winkler), according to the manufacturers' instructions. RNA concentration and integrity were assessed using a Qubit 4 Fluorometer (Thermo Fisher Scientific) with RNA quantification and RNA IQ assays, respectively. Library construction was performed at Macrogen Inc. facilities (Seoul, Republic of Korea) using the TruSeq kit (Illumina). mRNA libraries were then sequenced on the Illumina NovaSeq platform using 2 × 100 bp paired‐end reads.

### 2.3 Read Processing and Differential Gene Expression Analysis

The paired‐end reads obtained were quality‐filtered using fastp v0.23.1 (Chen et al. 2018) with a minimum base quality threshold of Q20 (−q 20), and adapters were removed using Cutadapt v1.18 (Martin 2011) with default parameters. Filtered reads were mapped to the corresponding reference genome using STAR v2.7.9a (Dobin et al. 2012) with coordinate‐sorted BAM output option (−outSAMtype BAM SortedByCoordinate). Alignments were filtered using SAMtools v1.13 (Danecek et al. 2021), retaining paired alignments with a mapping quality score ≥ 30 (−q 30) and properly paired reads (−f 2), and paired‐end reads were synchronized using Fastq‐Pair v0.4 (Edwards and Edwards 2019) with default parameters. Gene‐level read counts were obtained using featureCounts v2.0.1 (Liao et al. 2014) with paired‐end counting (−p), and unstranded libraries (−s 0). Differential expression analysis was performed following the standard edgeR workflow described in the edgeR user guide, including library‐size normalization using the trimmed mean of M‐values (TMM), dispersion estimation, and differential expression testing using the exact test (Robinson et al. 2010). The following three criteria were applied to select differentially expressed genes: (i) |logFC| > 1, (ii) *p*‐value < 0.001, and (iii) FDR < 0.05. MDS plots were generated to evaluate sample clustering. The complete R script used for differential expression analysis and MDS visualization is provided in Text S1.

### 2.4 Protein–Protein Interaction Networks Analysis and Hub Gene Detection

To visualize and explore protein–protein interaction (PPI) networks among DE gene lists, Cytoscape v3.10.4 (Killcoyne et al. 2009) was used. PPI information for each dataset was retrieved using the STRING database plugin v2.2.0 (Doncheva et al. 2023), and network structural parameters, including network density (ND), average path length (APL), and clustering coefficient (CC), were estimated from the resulting networks. The CytoHubba plugin v0.1 (Chin et al. 2014) was then used to identify the top 20 hub genes of each PPI network based on degree and betweenness centrality. Module detection was performed using the GLay algorithm in Cytoscape, which identifies densely interconnected groups of nodes within the network. Modules were visualized using distinct colors, with each color representing a different detected module. It is important to note that zebrafish STRING annotations were used for PPI network inference in *O. ascotanensis* .

### 2.5 Functional Enrichment of Gene Lists

Gene set enrichment analysis was performed with the STRING database plugin v2.2.0 on Cytoscape to reveal Gene Ontology (GO) terms or KEGG pathways enriched, using a cutoff *p*‐value = 0.001 and FDR < 0.05 as selection criteria. Enrichment results were visualized in dotplots using the R packages ggplot2 (Wickham 2011), enrichplot v1.31.4 (Yu 2026), and DOSE (Yu et al. 2015).

### 2.6 Ortholog Inference

To validate PPI species comparison, orthologs inference analysis was performed with OrthoFinder 2 v2.5.4 (Emms and Kelly 2019), using as input the 57,188 CDS of *D. rerio* Tuebingen genome version (Accession number PRJNA1193077) and the 33,485 CDS of *O. ascotanensis* genome (Accession number PRJNA293101). Orthogroup‐assigned CDS and the number and type of orthogroups were reported.

## 3 Results

### 3.1 Does UVB Tolerance Affect Transcriptomic Magnitude?

After read processing, we kept, on average, 15.5 M reads uniquely‐mapped to genes per sample, and a gene count matrix was built. To explore sample similarities, multidimensional scaling (MDS) analyses were performed, and plots were generated for the three experimental groups conforming to a UVB tolerance gradient for fish, namely, zebrafish WT (Figure S1A), zebrafish *nacre* mutants (Figure S1B), and *Orestias ascotanensis* (Figure S1C). As can be observed, sample clustering showed a pattern consistent with an effect of UVB radiation in all experimental groups. Although biological replication was limited, consistent patterns across individuals supported exploratory comparisons among the three models.

We then performed a differential expression analysis to detect differentially expressed (DE) genes in the three control and treatment experimental groups. Remarkably, the transcriptomic magnitudes, defined as the number of DE genes, agree with the UVB tolerance gradient proposed, being WT zebrafish the lowest response with 623 DE genes (390 up‐regulated, and 233 down‐regulated), followed by the melanin‐lacking mutant line (*nacre*) with an intermediate transcriptomic magnitude of 768 DE genes (212 up‐regulated, and 556 down‐regulated), and *O. ascotanensis* , having the highest transcriptomic magnitude with 4540 DE genes (for instance, 2832 up‐regulated, and 1708 down‐regulated) (Tables [Link], [Link], [Link], respectively). Despite the same UVB exposure treatment, the transcriptomic magnitude of *O. ascotanensis* was 6.5 times higher than that of zebrafish lines, consistent with an enhanced transcriptional responsiveness to UVB exposure. Furthermore, by normalizing the number of DE genes as a fraction of the total gene number per species (30,895 genes in *Danio rerio* , and 21,024 genes in *O. ascotanensis* ), WT zebrafish showed DE changes in approximately 2% of the analyzed annotated genes, *nacre* in 2.4%, and *O. ascotanensis* in 21.6%. Thus, *O. ascotanensis* showed a substantially higher proportion of annotated genes classified as differentially expressed than either zebrafish line. However, this proportion should be interpreted with caution because differences in genome annotation completeness between the model zebrafish genome and the less extensively annotated *O. ascotanensis* genome may affect the denominator used for this calculation. Importantly, the difference in absolute numbers of DE genes remained evident, with 4540 DE genes detected in *O. ascotanensis* compared with 623 and 768 in WT and *nacre* zebrafish, respectively. It is also important to note that in *nacre* mutants, despite a slight increase in transcriptomic magnitude and in the proportion of the genome being modulated in comparison to WT zebrafish, the increment in DE genes is mainly explained by the large number of down‐regulated genes, consistent with transcriptomic dysregulation due to the absence of melanin protective pigment.

Next, to explore the relationships among DE genes for all transcriptomic responses, a Venn diagram was built (Figure S1D). Interestingly, only 20 genes were differentially expressed across all experimental groups, whereas most DE genes were unique to each model (388 in WT, 524 in *nacre*, and 4453 in *O. ascotanensis* ). This pattern indicates that UVB responses are largely condition‐specific, with relatively little overlap in gene identity across the proposed tolerance gradient. However, because gene expression is organized within gene regulatory networks (GRNs), differences among responses may arise not only from the identity of DE genes, but also from how those genes are organized and interact.

### 3.2 Network Reorganization Across the UVB Tolerance Gradient

Transcriptomic magnitude alone does not fully capture the biological responses to UVB exposure, highlighting the importance of examining transcriptomic architecture. Therefore, PPI networks were used to explore transcriptomic responses at a higher level of organization, allowing the identification of connectivity patterns, network modules, and highly connected genes.

**FIGURE 1:**
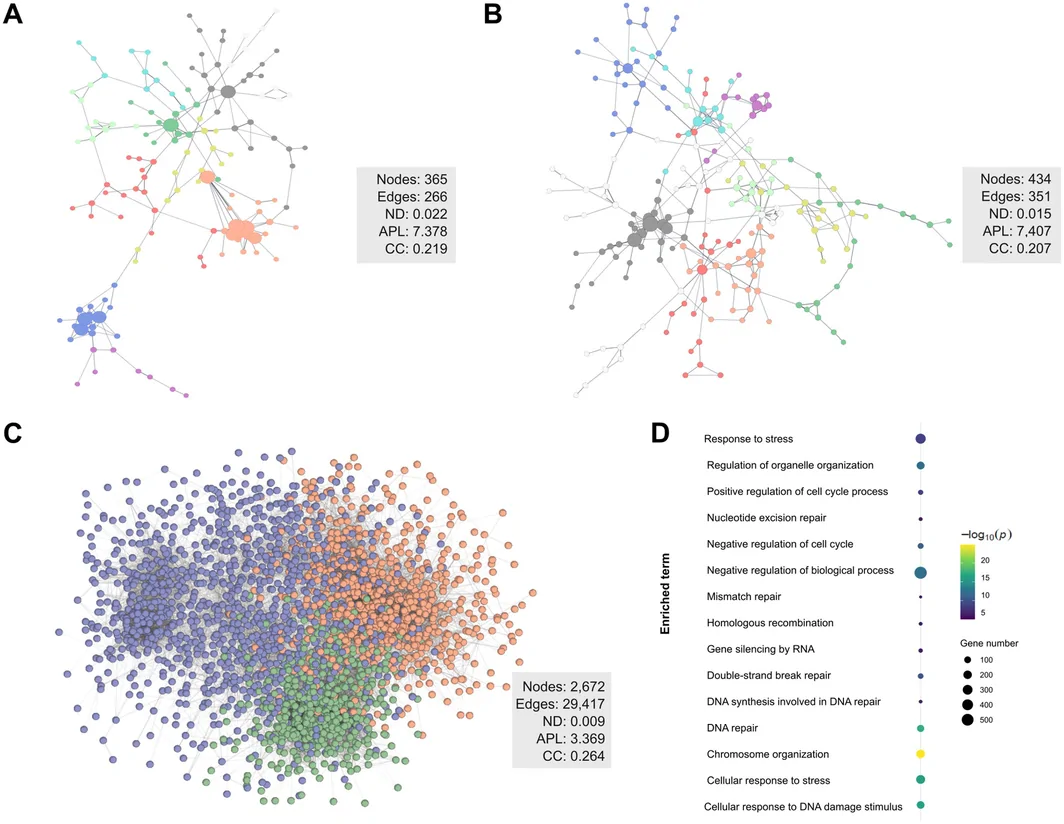
Network‐based analysis of transcriptomic responses across the UVB tolerance gradient. Protein–protein interaction (PPI) networks were inferred from differentially expressed (DE) genes identified after UVB exposure. For zebrafish networks, only the largest connected component is shown. Nodes are colored according to GLay community membership and scaled according to degree centrality. Network density (ND), average path length (APL), and clustering coefficient (CC) are indicated for each complete network. (A) PPI network inferred from DE genes in wild‐type zebrafish. (B) PPI network inferred from DE genes in the melanin‐deficient zebrafish mutant *nacre*. (C) Three largest modules identified in the PPI network inferred from DE genes in the UVB‐adapted fish *Orestias ascotanensis* . (D) Functional enrichment analysis of DE genes. As no significantly enriched categories were detected in either zebrafish line, only enriched categories identified in *O. ascotanensis* are shown. Dot size represents the number of genes associated with each category, whereas color indicates the enrichment ‐log10(p).

PPI networks inferred from DE genes differed markedly across the proposed UVB tolerance gradient (Figure 1). Consistent with the differential expression analyses, WT and *nacre* zebrafish networks were considerably smaller than the *O. ascotanensis* network. However, differences extended beyond transcriptomic magnitude. The zebrafish networks were characterized by relatively small and weakly connected modules, whereas the *O. ascotanensis* network displayed large interconnected modules forming an extensive network architecture (Figure 1A–C). Structural metrics supported these differences. WT and *nacre* networks exhibited low edge‐to‐node ratios (0.73 and 0.81, respectively), indicating sparse connectivity. In contrast, the *O. ascotanensis* network showed an edge‐to‐node ratio of 11.01, reflecting substantially greater connectivity among DE genes.

Because WT and *nacre* networks contained comparable numbers of nodes, their structural properties were compared to evaluate the potential contribution of melanin to UVB responses. The WT network displayed the highest network density and a relatively high average path length, suggesting the presence of compact local clusters with limited global interconnectivity. In contrast, the *nacre* network exhibited lower network density and the lowest clustering coefficient, consistent with a more diffuse and less clustered architecture. These differences suggest that the absence of melanin is associated with changes in the organization of transcriptomic responses to UVB exposure. Conversely, the *O. ascotanensis* network combined the highest clustering coefficient with the lowest average path length, indicating a highly interconnected architecture composed of densely connected modules. Together, these structural features were consistent with the proposed UVB tolerance gradient, with WT zebrafish displaying a contained architecture, *nacre* exhibiting a more diffuse architecture, and *O. ascotanensis* showing a highly interconnected and modular transcriptomic response.

To evaluate the functional specificity associated with these network architectures, enrichment analyses were performed using DE genes represented in each PPI network (Figure 1D). No significantly enriched categories were detected in either zebrafish line. In contrast, *O. ascotanensis* showed significant enrichment for pathways associated with DNA repair, cell‐cycle regulation, and responses to cellular stress. These results indicate that the extensive network organization observed in *O. ascotanensis* is accompanied by greater functional specialization, whereas zebrafish responses remained functionally diffuse.

**FIGURE 2:**
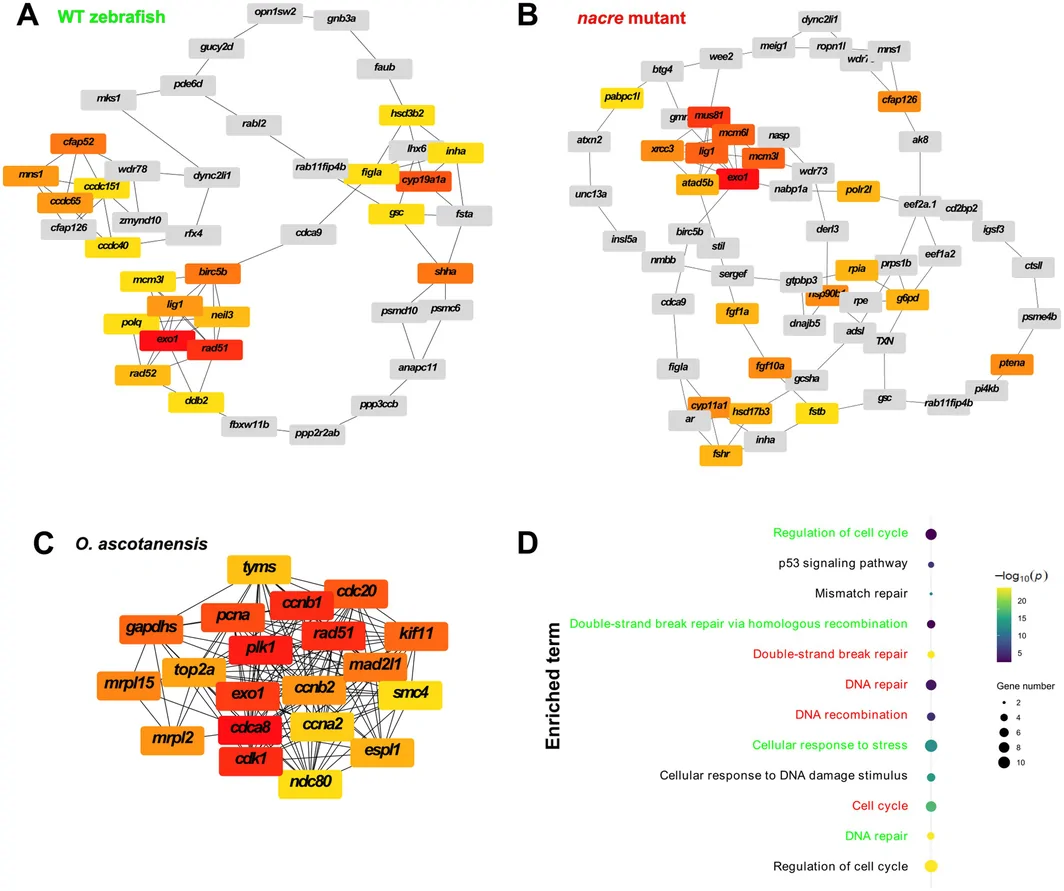
Network‐based analysis of highly connected (degree) DE genes per experimental groups. (A) PPI network inferred from the top 20 degree‐based hub genes detected for WT zebrafish. Node color indicates the ranking position, with red being the top‐ranked node. Gray nodes are included only to give biological context due to the low connectivity among DE genes. (B) PPI network inferred from the top 20 degree‐based hub genes detected for the *nacre* mutant. Node color indicates the ranking position, with red being the top‐ranked node. Gray nodes are included only to give biological context due to the low connectivity among DE genes. (C) PPI network inferred from the top 20 degree‐based hub genes detected for *O. ascotanensis* . Node color indicates the ranking position, with red being the top‐ranked node. (D) Dotplot representing the functional enrichment analysis performed in degree‐based hub genes detected for each experimental group. Term color indicates the experimental group where the category was enriched. For instance, WT in green, *nacre* in red, and *O. ascotanensis* in black. Dot size is related to gene number in the enriched category, while color indicates the associated ‐log10(p).

To further explore the organization of transcriptomic responses, hub genes were identified using degree and betweenness centrality measures. Networks constructed from the top 20 degree‐based hub genes revealed contrasting patterns among experimental groups (Figure 2A–C). Both zebrafish lines displayed relatively small and clustered hub networks, whereas *O. ascotanensis* exhibited a compact and highly interconnected hub architecture. Despite these differences, only one degree‐based hub gene, exonuclease 1 (*exo1*), was shared across all three groups. Additional shared genes included *rad51* between WT and *O. ascotanensis* , and *mcm3l* and *lig1* between WT and *nacre*. Many of the identified hub genes are associated with processes relevant to UVB responses, including DNA repair, cell‐cycle regulation, and cellular stress responses.

Functional enrichment analyses performed on degree‐based hub genes recovered pathways associated with DNA replication, DNA repair, homologous recombination, mismatch repair, nucleotide excision repair, and *p53* signaling (Figure 2D). In contrast, betweenness‐based hub genes did not recover significantly enriched categories in any experimental group (Figure S2A–C). This pattern suggests that degree centrality more effectively captures the functional organization of transcriptomic responses than betweenness centrality. Together, these results indicate that transcriptomic responses differ not only in magnitude but also in their organization, connectivity, and functional specialization across the proposed UVB tolerance gradient.

### 3.3 Conserved Background and Transcriptomic Reorganization

To facilitate cross‐species comparisons at the network level, we first performed an ortholog inference analysis. A total of 86.0% of zebrafish CDS and 73.1% of *O. ascotanensis* CDS (49,178 and 24,479 CDS, respectively) were assigned to orthogroups, indicating substantial conservation between both fish genomes. Orthogroup relationships included many‐to‐many (1572:1174), one‐to‐many (1310:472), many‐to‐one (6067:1359), and one‐to‐one orthologs (1080:1080). These one‐to‐one orthologs provided a conserved evolutionary framework for comparative network analyses.

**FIGURE 3:**
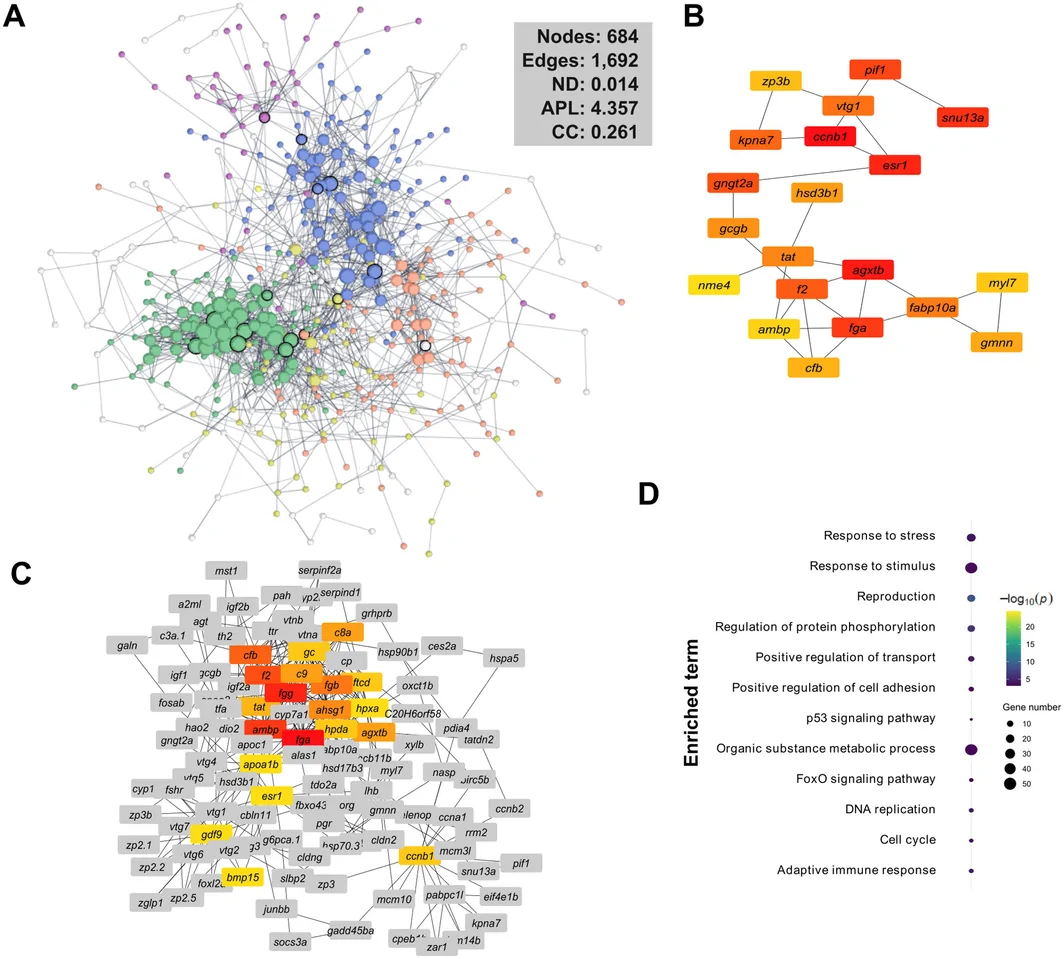
Network‐based analysis performed on one‐to‐one orthologs shared between zebrafish and *O. ascotanensis* . (A) PPI network inferred from the one‐to‐one ortholog dataset shared between both fish species. Network structural parameters, including network density (ND), average path length (APL), and clustering coefficient (CC), are indicated. Nodes are colored according to GLay community membership, node size is proportional to degree centrality, and highlighted borders indicate genes identified as hubs by both degree and betweenness centrality analyses. (B) PPI network formed by the top 20 betweenness‐based hub genes identified from the 1:1 ortholog network. Node color indicates ranking position, with red corresponding to the highest‐ranked genes. (C) PPI network formed by the top 20 degree‐based hub genes identified from the 1:1 ortholog network. Node color indicates ranking position, with red corresponding to the highest‐ranked genes. Gray nodes are shown to provide network context due to the limited connectivity among hub genes. (D) Dot plot representing the functional enrichment analysis performed on the top 20 degree‐based hub genes identified from the 1:1 ortholog network. Dot size represents the number of genes associated with each category, whereas color indicates the corresponding ‐log10(p). No significantly enriched categories were recovered for betweenness‐based hub genes; therefore, only results from degree‐based hubs are shown.

Using the 1080 one‐to‐one orthologs shared between species, a PPI network was inferred, recovering 684 nodes and 1692 edges (Figure 3A). Community detection analysis revealed a modular architecture composed of several interconnected functional groups. Hub genes identified using betweenness (Figure 3B) and degree (Figure 3C) centrality highlighted a subset of highly connected genes occupying central positions within the network. Functional enrichment analyses performed on the complete network and on the top degree‐based hub genes recovered broad biological processes, including reproduction, regulation of protein phosphorylation, response to stress, cell cycle regulation, and DNA replication (Figure 3D). In addition, enrichment of the *p53* signaling pathway among degree‐based hub genes suggests conservation of core genome‐maintenance functions within the ortholog dataset. Together, these results indicate that the 1:1 ortholog network captures a broadly conserved functional background shared between zebrafish and *O. ascotanensis* .

Within this conserved framework, differential expression analyses identified 71 ortholog genes that were differentially expressed in *O. ascotanensis* , compared with 24 in WT zebrafish, 19 in *nacre* mutants, and only 4 shared across all experimental groups. These results indicate that only a small fraction of the conserved ortholog repertoire is involved in UVB responses, and that distinct subsets of conserved genes are engaged across the proposed UVB tolerance gradient.

Comparison of the ortholog network with the *O. ascotanensis* DE network revealed notable differences in network organization and functional specialization. Whereas the ortholog network was characterized by broad and relatively unspecific biological functions, the *O. ascotanensis* network exhibited stronger functional enrichment associated with DNA repair, cell‐cycle regulation, and cellular stress responses. These observations are consistent with the idea that adaptive responses emerge through the differential organization of a conserved functional background rather than through entirely novel biological processes. In this context, the *O. ascotanensis* transcriptomic response appears as a more specialized configuration of conserved functional components, supporting the hypothesis that differences across the proposed UVB tolerance gradient are associated with alternative modes of transcriptomic organization.

### 3.4 Adaptive Signals Are Associated With Highly Interconnected Transcriptomic Networks

Our results are consistent with adaptive responses to UVB radiation in *O. ascotanensis* , reflected in both transcriptomic organization and signatures of positive selection. Whereas RNA‐seq analyses capture short‐term transcriptional responses to UVB exposure, previous dN/dS analyses identified genes exhibiting signatures of long‐term evolutionary change, including genes associated with DNA repair pathways (di Genova et al. 2022). Integrating these complementary datasets provides an opportunity to explore the relationship between transcriptional responses and evolutionary signatures in this high‐altitude fish.

**FIGURE 4:**
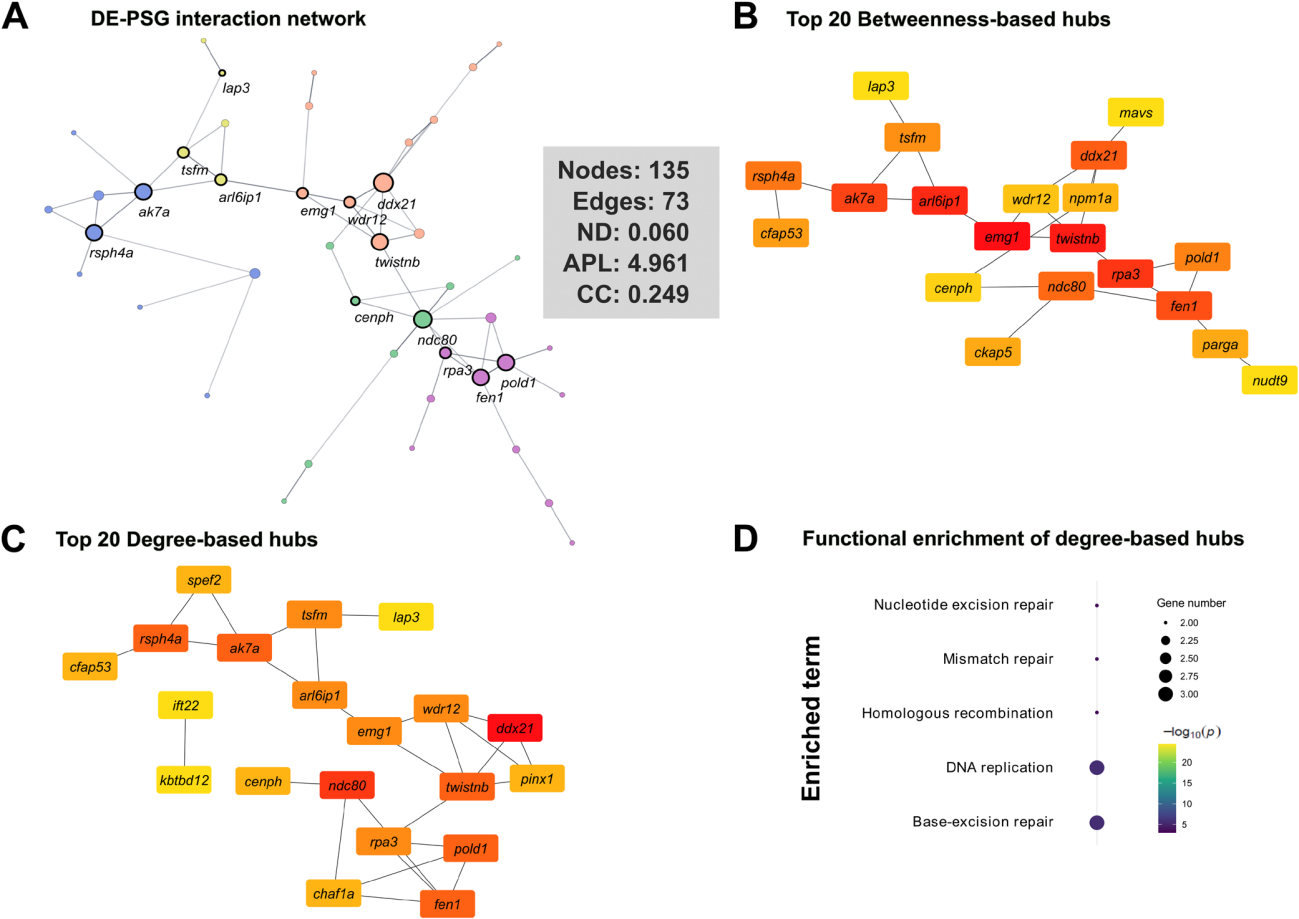
Network‐based analysis of differentially expressed (DE) and positively selected genes (PSG) in *O. ascotanensis* . (A) Largest connected component of the PPI network constructed from genes that were both differentially expressed and under positive selection in *O. ascotanensis* . Network structural parameters estimated from the complete network, including network density (ND), average path length (APL), and clustering coefficient (CC), are indicated. Nodes are colored according to GLay community membership, node size is proportional to degree centrality, and highlighted borders indicate genes identified as hubs by both degree and betweenness centrality analyses. (B) Subnetwork formed by the top 20 genes ranked by betweenness centrality. Node color indicates ranking position, with red corresponding to the highest‐ranked genes. (C) Subnetwork formed by the top 20 genes ranked by degree centrality. Node color indicates ranking position, with red corresponding to the highest‐ranked genes. (D) Dot plot showing functional enrichment analyses performed on the top 20 degree‐ and betweenness‐based hub genes. As no significantly enriched categories were recovered for betweenness‐based hubs, only enriched terms associated with degree‐based hubs are shown. Dot size represents the number of genes associated with each category, whereas color indicates the corresponding ‐lo10(p).

To investigate this relationship, RNA‐seq data were integrated with the dN/dS analyses previously conducted in *O. ascotanensis* . Functional enrichment analysis of the 782 positively selected genes with known annotation did not recover significantly enriched categories. However, overlap analyses revealed that 158 positively selected genes were also differentially expressed in *O. ascotanensis* following UVB exposure, compared with only 19 of the 71 one‐to‐one DE orthologs shared between species. A PPI network constructed from these DE and positively selected genes contained 135 nodes and 73 edges, resulting in an edge‐to‐node ratio of 0.54 (Figure 4A). Community detection analyses identified five modules within the largest connected component, indicating that genes simultaneously exhibiting differential expression and signatures of positive selection were organized into interconnected network modules. Network structural metrics, including high network density (ND) and clustering coefficient (CC) together with a moderate average path length (APL), were consistent with a densely connected and modular architecture.

To further characterize the organization of this network, hub genes were identified using degree and betweenness centrality measures. Similar sets of genes emerged from both approaches (Figure 4B,C), with 15 hub genes shared between degree‐ and betweenness‐based rankings. Several of these highly connected genes occupied central positions within the network modules identified in Figure 4A, suggesting that connectivity patterns are concentrated within specific regions of the DE‐positively selected network.

Functional enrichment analyses performed on the top 20 degree‐ and betweenness‐based hub genes revealed contrasting patterns. No significantly enriched categories were recovered for betweenness‐based hubs, whereas degree‐based hubs were enriched in pathways related to DNA replication, nucleotide excision repair, mismatch repair, homologous recombination, and base‐excision repair (Figure 4D). These results suggest that degree centrality more effectively captures the functional organization of the DE‐positively selected network than betweenness centrality.

Finally, integrating differential expression and positive selection revealed that genes carrying signatures of both processes were concentrated within a highly interconnected network enriched for genome maintenance functions. Several highly connected genes, including *ddx21*, *emg1*, and *wdr12*, are associated with nucleolar activity and ribosome biogenesis, processes increasingly recognized as being linked to genome maintenance and cellular stress responses. The co‐occurrence of DNA repair genes and nucleolar‐associated genes within the same interconnected network suggests coordinated organization among functional modules involved in genome maintenance. Together, these findings indicate that genes associated with both differential expression and positive selection are not randomly distributed across the transcriptome, but are organized within interconnected functional networks linked to DNA repair, cellular stress responses, and genome maintenance.

Overall, our findings show that adaptive differences in UVB response are not explained by transcriptomic magnitude alone, but emerge from the differential organization and functional specificity of a conserved transcriptomic architecture across the UVB tolerance gradient.

## 4 Discussion

Here, we show that transcriptomic responses to UVB are organized along a gradient spanning physiological protection, increased susceptibility, and evolutionary adaptation, each associated with distinct modes of transcriptomic architecture.

### 4.1 From Gene Lists to Network Architecture

In the post‐genomic era, biological functions are no longer viewed as properties of individual genes, but rather as emergent outcomes of complex interactions among genes and molecules (Hartwell et al. 1999; Bray 2003; Alon 2003) organized within gene regulatory networks (GRNs). Consequently, network biology has emerged as a framework for understanding cellular and organismal organization from a systems perspective (Barabási and Oltvai 2004). To investigate phenotypic variation, transcriptomic analyses have provided a global view of changes in gene expression (Costa‐Silva et al. 2017), enabling the identification of responsive genes, regulatory pathways, and enriched biological functions.

Despite the power of transcriptomic approaches to identify differentially expressed genes and enriched pathways, these analyses remain largely centered on gene lists, limiting our ability to understand how adaptive responses emerge under environmental stress. Our results suggest that this perspective captures only part of the biological response to UVB exposure. Because genes operate within interconnected regulatory networks, differences among responses are reflected not only in the identity or number of differentially expressed genes but also in how those genes are organized within transcriptomic architectures. Consistent with this view, network‐level analyses revealed marked differences in organization and functional specificity across the UVB tolerance gradient, indicating that adaptation may involve the reorganization of regulatory systems rather than changes in isolated genes. Together, these findings indicate that understanding how organisms respond and adapt to environmental stress requires moving beyond gene lists toward the analysis of transcriptomic architecture.

### 4.2 A Conserved Functional Background Underlies UVB Response

Here, we examined a UVB tolerance gradient involving two phylogenetically divergent fish species (Betancur‐R et al. 2017), in which *O. ascotanensis* displayed a markedly stronger and more functionally specialized transcriptomic response than zebrafish. Despite these differences, ortholog‐based analyses revealed a shared functional framework between species. Transcriptomic magnitudes and functional specificities remained consistent with the proposed UVB tolerance gradient even within this reduced ortholog framework. These results suggest that the contrasting UVB‐responsive architectures observed across species are built upon conserved functional components, while adaptive divergence may emerge through their differential organization and specialization. Thus, adaptation appears to involve the reorganization of existing functional systems rather than the emergence of entirely novel transcriptomic functions.

### 4.3 Melanin as an Evolutionary Buffer

As a laboratory model system, zebrafish offers a wide range of transgenic and mutant lines. We therefore took advantage of the *nacre* mutant, deficient in the master regulator of melanocyte development MITF (Levy et al. 2006), to establish a melanization model for studying the protective role of this cellular pigment in response to UVB radiation, using the table 5 line as the wild‐type melanin‐producing control and the *nacre* mutant as a melanin‐lacking unprotected condition.

As a protective cellular pigment, melanin absorbs and dissipates photons from ionizing radiation within its molecular structure (Wolbarsht et al. 1981), in addition to performing a variety of ecological and biochemical functions that have been studied extensively, particularly in fungi (Casadevall et al. 2017). Beyond melanin synthesis itself, fish also mitigate UVB‐induced damage through the dynamic redistribution of melanin within melanosomes and chromatophore cell types (Mueller and Neuhauss 2014). Although the protective properties of melanin are well established at the molecular level, much less is known about how cellular responses to UVB radiation are organized in its absence.

Our results showed that, despite similar transcriptomic magnitudes and functional profiles, the *nacre* mutant exhibited a greater proportion of down‐regulated genes than wild‐type fish, suggesting increased transcriptomic disruption in the absence of melanin. The contrasting architectures observed between WT and *nacre* indicate that melanin reduces the transcriptomic disruption associated with UVB exposure, contributing to a more coordinated response architecture. These findings suggest that melanin functions not only as a physical barrier against UVB damage but also as a physiological buffer that stabilizes transcriptomic responses under environmental stress. By reducing the immediate cellular consequences of UVB exposure, such buffering effects may influence the selective pressures experienced by organisms in high‐radiation environments and shape the cellular context upon which adaptive responses evolve.

### 4.4 Adaptive Network Modules and DNA Repair

*O. ascotanensis* exhibited a transcriptomic response that differed markedly in magnitude, architecture, and functional specificity from those observed in zebrafish. Previous studies have proposed that genes and proteins do not evolve as isolated entities, but rather as components of integrated and modular networks (Fraser 2005), where highly connected elements often play central roles in biological function and organismal performance (Jeong et al. 2001). Consistent with this view, our analyses revealed that highly connected genes occupied central positions within functionally specialized transcriptomic networks, while genes showing both differential expression and signatures of positive selection were organized into interconnected network modules. These findings suggest that adaptation to UVB stress may involve the reorganization of coordinated molecular systems rather than changes in isolated genes.

From this perspective, transcriptomic components of phenotypic plasticity, here considered as short‐term transcriptional responses in contrast to long‐term evolutionary adaptation, should be evaluated not only in terms of transcriptomic magnitude, but also in terms of functional organization and specificity. Similar patterns have been reported in vertebrate adaptation to thermal stress, high‐altitude environments, and climate‐related environmental change (Storz et al. 2010; Rodríguez et al. 2017; Wollenberg Valero et al. 2021). In this context, our results indicate that adaptation may be manifested not only through changes in gene expression levels, but also through increased organization and functional specialization of transcriptomic architecture, reflected in the highly interconnected modules and specific functional enrichments observed in *O. ascotanensis* .

The network‐level adaptation hypothesis was further supported by the integration of evolutionary and transcriptional signals. Previous dN/dS analyses identified genes showing signatures of long‐term evolutionary change in *O. ascotanensis* , including genes associated with DNA repair pathways (di Genova et al. 2022). In the present study, 158 positively selected genes were also differentially expressed following UVB exposure. Although functional enrichment of the 782 positively selected genes with known annotation did not recover significant categories, the genes showing both positive‐selection signatures and differential expression were organized into a network containing five interconnected modules. This pattern suggests that evolutionary and transcriptional signals are not randomly distributed across the transcriptome, but may converge within specific regions of the network.

Functional enrichment of the degree‐based hubs within this DE‐positively selected network revealed pathways related to DNA replication, nucleotide excision repair, mismatch repair, homologous recombination, and base‐excision repair, whereas betweenness‐based hubs did not show significant enrichment. These results indicate that genome‐maintenance functions are concentrated among highly connected genes within the network integrating transcriptional and evolutionary signals. Moreover, several genes showing both differential expression and positive‐selection signatures, including *ddx21*, *emg1*, and *wdr12*, occupied central positions within the network and are associated with nucleolar activity and ribosome biogenesis, processes that may contribute to cellular stress responses and genome maintenance. Together, these findings suggest coordinated organization among functional components involved in genome maintenance rather than independent changes in individual genes.

The one‐to‐one ortholog analysis further showed that several functions identified in the *O. ascotanensis* response are shared with zebrafish, including DNA replication and *p53* signaling among degree‐based hubs. However, the *O. ascotanensis* DE network showed stronger enrichment for DNA repair, cell‐cycle regulation, and cellular stress responses. Thus, the adaptive signal identified here is better interpreted as differential organization and specialization of functional components within the UVB‐responsive network than as the emergence of entirely novel genome‐maintenance functions.

These findings are also consistent with previous work showing that evolutionary divergence can involve changes in gene expression regulation, including differences associated with cis‐ and trans‐regulatory effects (Romero et al. 2012; Stern and Orgogozo 2008; Signor and Nuzhdin 2018). Our results extend this perspective by suggesting that evolutionary change may be associated not only with differences in gene expression but also with the organization of responsive genes within interconnected transcriptomic networks. Evidence from non‐vertebrate systems further indicates that evolutionary change can involve the regulation and reuse of existing genetic components across diverse lineages, including insects, plants, and fungi (Rebeiz et al. 2011; Martin and Orgogozo 2013), while recent work in *Drosophila* has provided evidence for genetic and transcriptional contributions to UV tolerance and DNA repair (Titus‐McQuillan et al. 2025). Thus, changes in transcriptomic architecture may represent an important component of how functional systems are reorganized during adaptive evolution.

### 4.5 Evolutionary Implications

Our findings suggest that adaptation to environmental stress involves changes in transcriptomic organization in addition to changes in individual genes. Wild‐type zebrafish and *O. ascotanensis* differed markedly in transcriptomic magnitude and functional specificity, yet both exhibited more coordinated architectures than the melanin‐deficient *nacre* mutant. Together, these findings support the view that adaptation may emerge from the reorganization of conserved regulatory systems, highlighting transcriptomic architecture as an important level of biological organization through which organisms respond and adapt to environmental stress.

### 4.6 Environmental Relevance of the UVB Challenge

The UVB exposure used in this study was designed as a standardized experimental challenge rather than as a direct simulation of the natural daily UVB dose experienced by fish in the field. The irradiance used in the chamber (15 W/m^2^) was comparable in magnitude to that reported by Yang et al. (2014) for which transcriptional responses were detected in adult fish. In addition, field measurements conducted in the Ascotán area confirmed the high UV environment experienced by *O. ascotanensis* . These measurements, however, were obtained using a UVAB sensor (280–400 nm) and therefore cannot be directly converted into a natural UVB dose. Thus, the experimental exposure should be interpreted as a controlled and reproducible UVB challenge intended to reveal differences in transcriptomic organization among species, rather than as a quantitative reproduction of natural UVB exposure. This distinction is particularly relevant because exposure in aquatic environments depends on water depth, transparency, and other optical properties that influence the amount of UV radiation reaching the skin.

### 4.7 Limitations and Future Directions

Here, we used a transcriptomic and network‐level approach to evaluate the effects of adaptation on gene expression. However, working with natural populations of *O. ascotanensis* involved a relevant trade‐off between biological replication and conservation constraints associated with sampling a threatened endemic species. We therefore limited sampling to three adult females per experimental group. This low replication reduces statistical power for differential expression analysis and may limit the stability of sample‐level clustering analyses such as MDS. Thus, the present results should be interpreted as exploratory evidence of contrasting transcriptomic architectures rather than as definitive estimates of population‐level variation or effect sizes. Nevertheless, consistent patterns across biological replicates and the convergence of differential expression, network, functional enrichment, ortholog, and positive‐selection analyses provide complementary evidence supporting the main patterns identified here.

An additional consideration is that the experimental comparison involved laboratory‐maintained zebrafish and wild *O. ascotanensis* . Although *O. ascotanensis* were exposed in the field immediately after capture, thereby minimizing the potential effects of transportation and prolonged laboratory acclimation, we cannot completely exclude effects associated with differences in environmental history, husbandry conditions, or physiological state between the two experimental systems. Therefore, the observed interspecific differences should be interpreted as differences in UVB‐responsive transcriptomic architecture between these biological contexts, rather than as a direct estimate of species‐specific UVB sensitivity under identical lifelong environmental conditions.

Another potential limitation of our work is related to the use of the STRING database in a cross‐species model design. The amount of accumulated experimental evidence in zebrafish far exceeds the knowledge available in the non‐model fish *O. ascotanensis*, and the use of STRING zebrafish annotation to analyze *O. ascotanensis* transcriptomic response could lead to bias. However, the one‐to‐one ortholog analysis identified a conserved set of genes shared between both species, within which the major comparative patterns remained detectable. Nevertheless, our conclusions rely on network‐level and comparative inferences, and experimental validation will be necessary to further evaluate the proposed mechanisms. For instance, the *Orestias* genus has a wide latitudinal distribution in the Andes, and further species comparisons will be needed to evaluate whether the adaptive transcriptomic features identified here are intrinsic to *O. ascotanensis* or represent a common feature across this high‐altitude fish genus. Furthermore, to evaluate whether the adaptive transcriptomic architecture identified here emerged as a result of long‐term isolation in high‐altitude environments, genomic comparisons with the phylogenetically sister family Fluviphylacidae (Morales et al. 2024) will be required.

Similarly, differences in genome annotation completeness between zebrafish and *O. ascotanensis* may influence comparisons based on the proportion of annotated genes classified as differentially expressed. Therefore, the 21.6% estimate should be interpreted cautiously and is not intended as a direct measure of the proportion of the complete genome responding to UVB. The absolute number of DE genes and the comparative analysis restricted to one‐to‐one orthologs provide complementary evidence that is less dependent on differences in genome‐wide annotation.

Overall, our results support a model in which adaptation to environmental stress is encoded not only in gene content, but also in the organization and functional specificity of transcriptomic architecture. Within this framework, shared functional components can be differentially organized across physiological and evolutionary contexts, generating distinct adaptive outcomes from a common molecular framework. More broadly, these findings suggest that understanding how organisms respond and adapt to environmental challenges requires considering not only which genes are involved, but also how regulatory systems are organized to produce coordinated biological responses.

## Author Contributions

**Camilo Valdivieso:** conceptualization (lead), data curation (lead), formal analysis (lead), investigation (lead), supervision (supporting), visualization (lead), writing – original draft (equal), writing – review and editing (equal). **Miguel Luis Allende:** conceptualization (equal), formal analysis (equal), funding acquisition (lead), project administration (lead), resources (lead), supervision (lead).

## Funding

This work was supported by the Center for Genome Regulation Millennium Institute (ICN2021_044).

## Conflicts of Interest

The authors declare no conflicts of interest.

## Supporting information


**Figure S1:** Sample clustering and relationships among DE genes. (A) Multidimensional scaling (MDS) plot showing sample clustering in WT zebrafish. TC = WT control; TUV = WT UVB‐treated. (B) MDS plot showing sample clustering in the *nacre* mutant. NC = *nacre* control; NUV = *nacre* UVB‐treated. (C) MDS plot showing sample clustering in *O. ascotanensis* . OC = *O. ascotanensis* control; OUV = *O. ascotanensis* UVB‐treated. (D) Venn diagram showing the overlap among DE genes identified in each experimental group. Total numbers of DE genes per group are indicated in parentheses.

**Figure S2:** Top 20 betweenness‐based hub genes identified in the experimental groups. (A) Subnetwork formed by the top 20 betweenness‐based hub genes identified in WT zebrafish. Node color indicates ranking position, with red corresponding to the highest‐ranked genes. (B) Subnetwork formed by the top 20 betweenness‐based hub genes identified in the *nacre* mutant. Node color indicates ranking position, with red corresponding to the highest‐ranked genes. Gray nodes are shown to provide network context due to the limited connectivity among hub genes. (C) Subnetwork formed by the top 20 betweenness‐based hub genes identified in *O. ascotanensis* . Node color indicates ranking position, with red corresponding to the highest‐ranked genes. Gray nodes are shown to provide network context due to the limited connectivity among hub genes.

**Table S1:** DE genes detected in WT zebrafish in response to UVB radiation.
**Table S2:** DE genes detected in the *nacre* mutant in response to UVB radiation.
**Table S3:** DE genes detected in *O. ascotanensis* in response to UVB radiation.

**Data S1:** ece374298‐sup‐0004‐SupplementaryTextS1.R.

## Data Availability

Raw sequencing reads generated in this study have been deposited in the NCBI Sequence Read Archive (SRA) under BioProject accession PRJNA1454215. Differential expression results and other derived datasets are provided in the Tables S1–S3. The complete R script used for differential expression analysis and MDS visualization is provided in Text S1.
